# Effects of Three-Wavelength-Cut Lens on Cone Contrast Sensitivity

**DOI:** 10.3390/vision10040064

**Published:** 2026-09-01

**Authors:** Shuji Nakatsuka, Tomoya Handa, Yo Iwata, Hirotaka Ito, Tomohito Watanabe, Kumiko Mokuno

**Affiliations:** 1 Department of Ophthalmology, Kariya Toyota General Hospital, Kariya 448-8505, Japan; hirotaka.ito@toyota-kai.or.jp (H.I.); dreams.kemagazin@gmail.com (T.W.); kts-mokuno@katch.ne.jp (K.M.); 2 Graduate School of Medical Sciences, Kitasato University, Sagamihara 252-0373, Japan; thanda@kitasato-u.ac.jp (T.H.); iwatayo@kitasato-u.ac.jp (Y.I.); 3 Department of Orthoptics and Visual Science, School of Allied Health Sciences, Kitasato University, Sagamihara 252-0373, Japan

**Keywords:** wavelength-control lens, three-wavelength-cut lens, ColorDx CCT-HD, contrast sensitivity

## Abstract

The purpose of this study was to quantitatively evaluate the effects of a three-wavelength-cut (TWC) lens, a type of wavelength-control lens, on cone contrast sensitivity in healthy adults using ColorDx CCT-HD (KONAN MEDICAL, Inc.). TWC lenses selectively attenuate light at wavelengths below approximately 420 nm, as well as around 460 and 585 nm. Fifteen healthy adults (mean age, 39.7 ± 9.0 years) were examined under two conditions: full refractive correction alone and full refractive correction with the TWC lens. Cone contrast sensitivity scores corresponding to long-wavelength-sensitive cones (L-cones), middle-wavelength-sensitive cones (M-cones), and short-wavelength-sensitive cones (S-cones) were measured. The L-cone-axis score was treated as the primary outcome, with the M- and S-cone-axis analyses considered exploratory. Paired comparisons were supplemented with a two-way repeated-measures ANOVA using lens condition and cone axis as within-subject factors. The primary L-cone analysis showed a significantly higher contrast sensitivity score with the TWC lens than without it (*p* = 0.022). The repeated-measures ANOVA showed a significant main effect of lens condition (*p* = 0.024), but no lens condition × cone axis interaction (*p* = 0.858). These preliminary findings suggest an overall lens-associated change in cone-axis-specific contrast sensitivity, while providing no statistical evidence that the effect is selective to the L-cone axis.

## 1. Introduction

**Figure 1:**
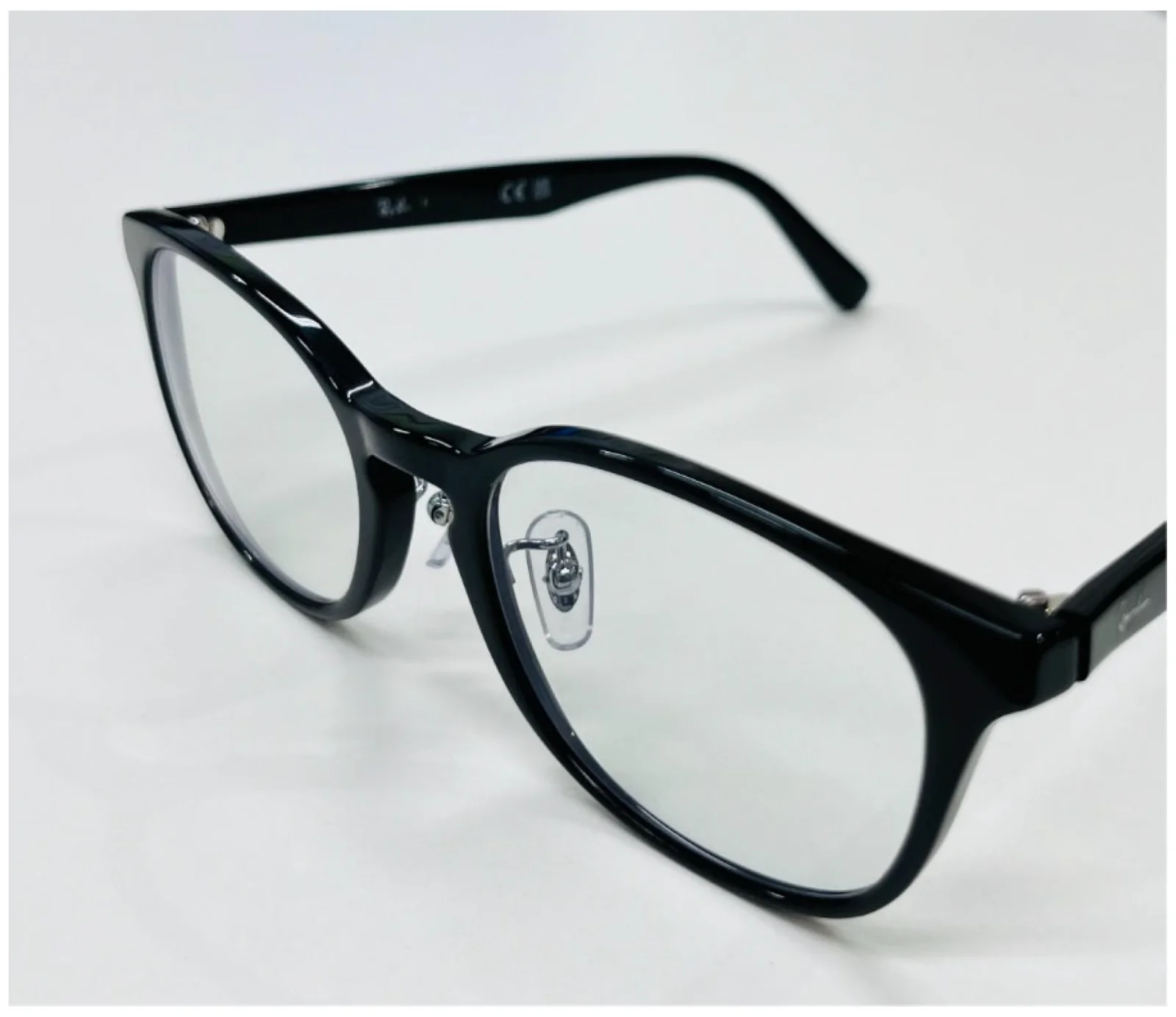
External appearance of the three-wavelength-cut lens (TWC lens). The TWC lens selectively attenuates specific wavelength bands and retains a nearly transparent, almost colorless appearance despite its wavelength-control properties.

Human color perception is based on the responses of three cone photoreceptor types in the retina: long-wavelength-sensitive cones (L-cones), middle-wavelength-sensitive cones (M-cones), and short-wavelength-sensitive cones (S-cones) [1]. These outputs are subsequently recoded by retinal ganglion cells and visual pathways into red–green (L−M), blue–yellow (S−(L+M)), and luminance (L+M) channels [2]. Color perception and cone contrast detection do not depend on the absolute response of each cone type, but rather on the relative differences in responses among the cone classes. Congenital color vision deficiency is one of the most common inherited visual disorders, affecting up to approximately 8% of males and 0.5% of females in some populations [3,4]. In addition, acquired color vision deficiency may occur with aging and in association with various ocular or neurologic disorders [5]. In recent years, wavelength-control lenses that selectively modulate specific visible light bands have been developed, and their potential to improve contrast sensitivity has been reported [6,7]. However, evidence regarding the effects of wavelength-filtering spectacle lenses on visual performance remains mixed, and further mechanism-oriented evaluations are needed [8]. In particular, yellow-cut lenses have been shown to improve luminance contrast by selectively attenuating short-wavelength light, thereby reducing light scatter [9]. Furthermore, differences in the cut rate around 585 nm have reportedly influenced the degree of contrast improvement [6], suggesting that wavelength-selective control conditions may affect visual function. The three-wavelength-cut lens (TWC lens; Hopnic Laboratory Co., Ltd., Sabae, Japan) is a wavelength-control lens designed to selectively attenuate three wavelength bands: below approximately 420 nm, around 460 nm, and around 585 nm [10]. The novelty of the TWC lens lies in its selective multi-band wavelength control, which attenuates specific short- and middle-wavelength regions while maintaining a relatively transparent appearance. TWC lenses are relatively new wavelength-control lenses, and publicly available data on their prevalence of use in clinical or daily settings are limited. At present, these lenses appear to be used mainly in research settings and selected practical applications aimed at improving visual quality. The external appearance of the TWC lens is shown in Figure 1. This lens was developed to improve contrast sensitivity while maintaining an almost colorless, transparent appearance. The proposed optical–psychophysical model underlying this lens technology is that selective attenuation of short-wavelength light may reduce intraocular light scatter and glare, whereas attenuation around 585 nm may alter the relative spectral excitations of the L- and M-cones, owing to the substantial overlap in their spectral sensitivities [2,11,12,13,14,15]. This model does not posit a direct modification of cone physiology; rather, it predicts that wavelength-selective filtering changes the spectral stimulus reaching the retina and thereby may alter measured cone-axis-specific contrast sensitivity. Because transmittance in the longer-wavelength range is relatively preserved, we hypothesized that TWC lens wear might produce a measurable change in cone-axis-specific contrast sensitivity under the present filtered-stimulus conditions, particularly along the L-cone axis. ColorDx CCT-HD is an instrument that allows quantitative evaluation of cone-axis-specific color contrast sensitivity [16,17]. However, measurements along individual cone-isolating axes are not equivalent to a direct assessment of postreceptoral L−M opponency, which would require chromatic modulation along an L−M opponent direction in color space [13,14]. Thus, ColorDx CCT-HD may provide a standardized, quantitative psychophysical method for evaluating the effects of wavelength-control lenses on cone-axis-specific contrast sensitivity [18]. Theoretically, the selective wavelength control of the TWC lens may alter cone-mediated visual signals; however, the mechanisms underlying these effects remain unclear. Previous studies on wavelength-control lenses, including TWC lenses, have reported beneficial effects on visual quality and contrast sensitivity. However, the specific cone-axis-dependent effects of these lenses remain insufficiently characterized. In particular, although cone contrast sensitivity has been examined in broader assessments of visual function, it remains unclear whether TWC lens wear produces a selective effect on specific cone pathways under a simple within-subject comparison. Clarifying this point is important because the spectral transmittance profile of the TWC lens may not influence all cone-mediated channels equally. Therefore, the present study was designed to focus specifically on cone contrast sensitivity along the L-, M-, and S-cone axes using ColorDx CCT-HD, with the aim of providing a more mechanism-oriented evaluation of the visual effects of TWC lens wear. The primary hypothesis tested in this study was whether TWC lens wear altered L-cone-axis contrast sensitivity, based on the spectral transmittance characteristics of the TWC lens and the optical and psychophysical working hypothesis described above. The M- and S-cone-axis analyses were considered exploratory secondary analyses. Accordingly, the purpose of this exploratory study was to investigate whether TWC lens wear is associated with changes in measured psychophysical contrast sensitivity along the L-, M-, and S-cone axes in healthy adults under filtered-stimulus conditions.

## 2. Materials and Methods

This study was designed as an exploratory pilot study that included 15 healthy adult participants. The study population consisted of healthy volunteers rather than patients. The mean age was 39.7 ± 9.0 years, the mean corrected visual acuity was logMAR −0.07 ± 0.09, and the mean refractive error was −2.92 ± 2.30 diopters (D). All 15 participants completed both testing conditions and were included in the final analysis. No participants were excluded after enrollment, and no missing data were observed. This was a non-randomized, within-subject study, and neither the participants nor the examiners were masked to the lens condition.

**Figure 2:**
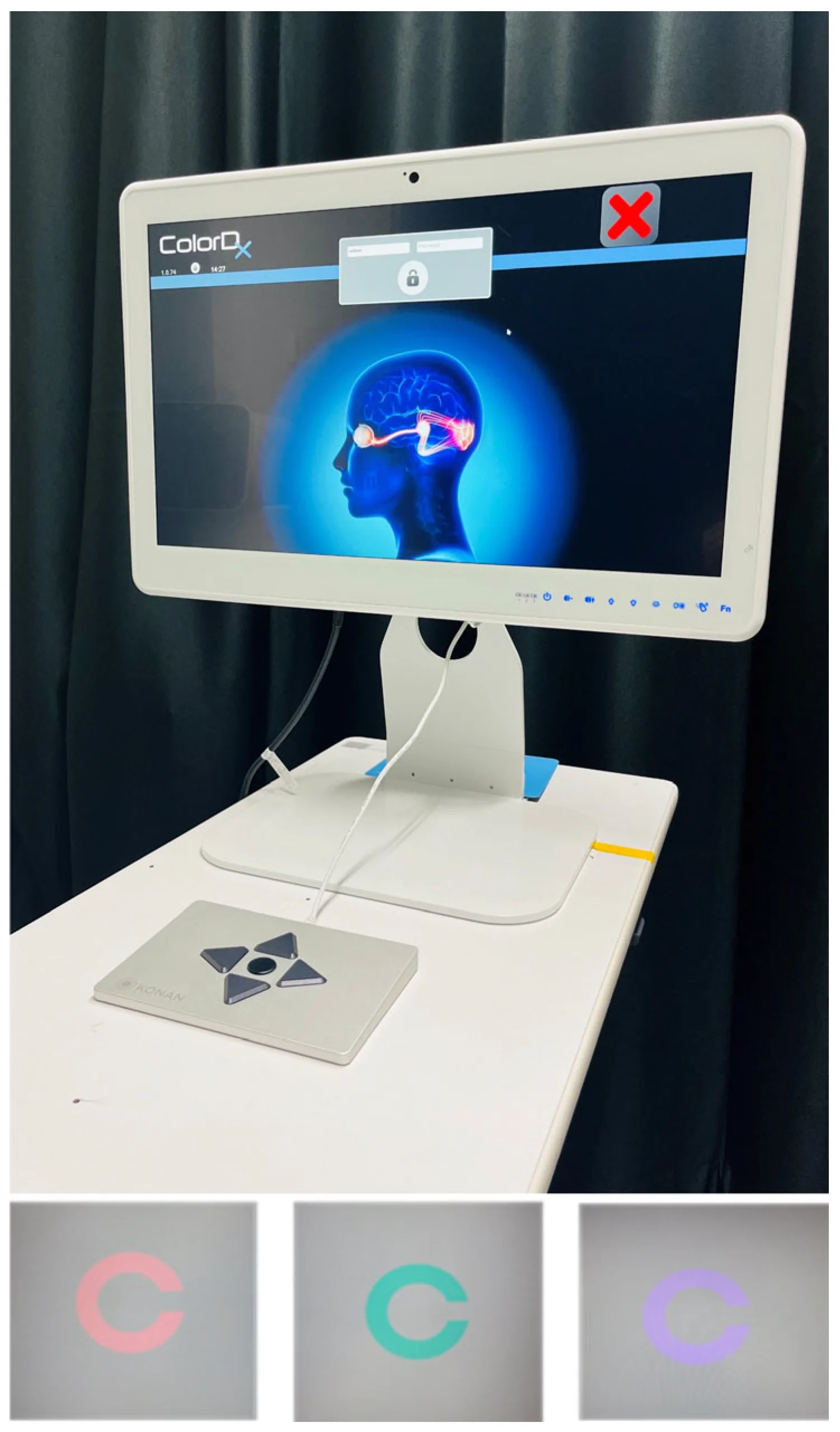
External appearance of the ColorDx CCT-HD system (KONAN MEDICAL, Inc.) and representative stimulus used for cone contrast sensitivity testing. The ColorDx CCT-HD is a computer-based cone contrast sensitivity testing system designed to quantify visual sensitivity along the L-, M-, and S-cone axes. The lower panel shows a representative Landolt ring stimulus presented on a neutral gray background. Participants were asked to identify the orientation of the gap in the Landolt ring, and cone contrast sensitivity scores were estimated from their responses using the device-guided procedure. The lower panel shows representative Landolt ring stimuli for the L-, M-, and S-cone axes (left to right) on a neutral gray background.

Color vision deficiency was screened using Ishihara pseudoisochromatic plates because the purpose of color vision testing in this exploratory study was to exclude participants with apparent red–green color vision deficiency, rather than to classify or quantitatively characterize subtle color vision abnormalities [19,20]. None of the participants showed evidence of color vision deficiency. Cone contrast sensitivity was measured using a ColorDx CCT-HD (Konan Medical USA, Inc., Irvine, CA, USA) (Figure 2). ColorDx CCT-HD is a computer-based cone contrast sensitivity testing system designed to quantify contrast sensitivity along the L-, M-, and S-cone axes. The device is based on the cone contrast test (CCT), which presents calibrated chromatic stimuli that selectively modulate contrast along specific cone axes to estimate cone-specific visual sensitivity [18].

In this study, ColorDx CCT-HD was used as the primary psychophysical measurement system to quantify changes in contrast sensitivity along the L-, M-, and S-cone axes between the conditions with and without the TWC lens, rather than to diagnose or classify color vision deficiency. A four-alternative forced-choice task, wherein participants identify the orientation of the gap in a Landolt ring presented on a neutral gray background, was performed. The stimulus contrast is adjusted along the L-, M-, or S-cone axis, and the threshold is estimated from the participant’s responses using the device-guided procedure. The results are displayed as cone contrast sensitivity scores, with higher scores indicating greater contrast sensitivity along the corresponding cone axis. The CCT method has been described and evaluated in previous peer-reviewed studies [18,21]. According to the manufacturer’s product information, the ColorDx CCT-HD system is FDA-listed and CE-marked [22], and the Japanese product information provides medical device notification numbers for the CCT-HD20 and CCT-HD22 models [23]. The manufacturer also states that ColorDx CCT-HD devices are precision-calibrated during manufacturing and include a USB colorimeter for periodic verification and calibration [22]. The test distance was set to 0.6 m, according to the default setting of the device. ColorDx CCT-HD provides two testing modes: an adaptive method and a full threshold method. In this study, the adaptive method was selected to shorten the examination time and reduce participant burden. In the adaptive procedure, the minimum number of stimuli required to estimate the threshold was presented sequentially for each cone type.

**Figure 3:**
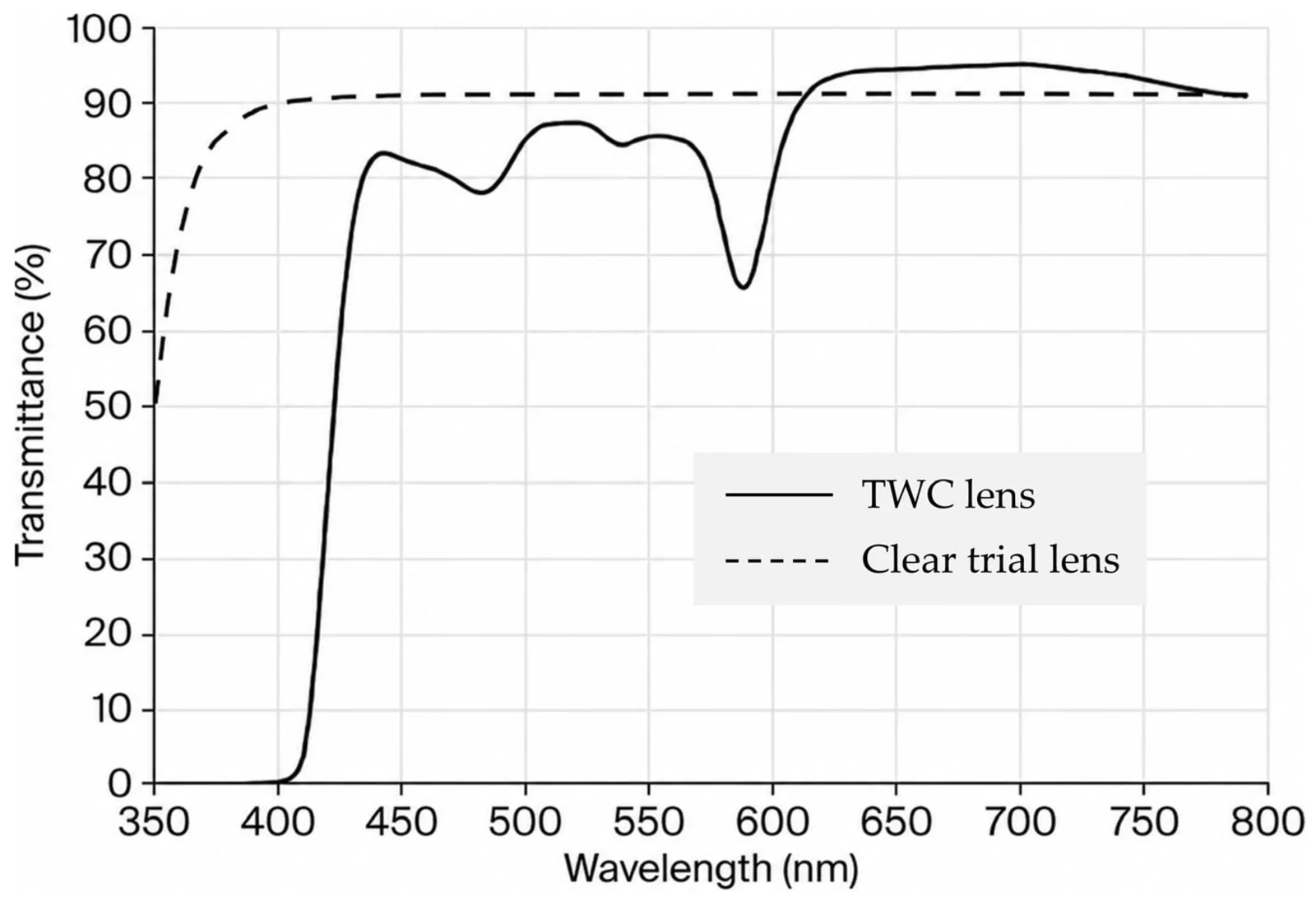
Spectral transmittance of the three-wavelength-cut lens (TWC lens) and a representative clear glass trial lens used for full refractive correction. The solid line represents the TWC lens, and the dashed line represents the clear glass trial lens. The TWC lens selectively attenuates light in the short-wavelength regions below approximately 420 nm and around 460 nm, as well as in the wavelength region around 585 nm, while the clear glass trial lens shows relatively uniform high transmittance across most of the visible wavelength range.

The testing sequence was predetermined using the device settings and was performed in the following order: L-cone, M-cone, and S-cone. Two test conditions were examined using a trial frame: full refractive correction with clear trial lenses (hereafter referred to as the without TWC lens condition) and the same full refractive correction with an additional TWC lens (hereafter referred to as the with TWC lens condition). The clear trial lenses and the TWC lens were visually similar in appearance. The measurements were performed binocularly. All examinations were conducted by two researchers (S.N. and T.W.). The participants’ subjective responses were entered into the device according to the device-guided procedure. The spectral transmittance profiles of the TWC lens and a representative clear glass trial lens used for full refractive correction are shown together in Figure 3. The testing order was assigned according to the participant enrollment number. The participants with odd-numbered registrations were tested first without the TWC lens, followed by those with the TWC lens, whereas those with even-numbered registrations were tested in the reverse order. In the TWC lens condition, a 1 min adaptation period was provided after lens wear before testing. The display of the testing device was a 22-inch monitor with a resolution of 1920 × 1080 pixels, refresh rate of 59 Hz, and luminance of 102.6 cd/m^2^. The room was illuminated using ceiling-mounted linear LED lamps (LFIR040 16N50D; DAEDUCK GDS Co., Ltd., Ansan, Republic of Korea; nominal color temperature, 5000 K). The study was conducted under indoor lighting conditions, with an illuminance of approximately 500 lx and a color temperature of approximately 4600 K, as measured using an illuminance meter (CL-200, Konica Minolta, Inc., Tokyo, Japan). The spectral power distribution of the ambient illumination and display, particularly in the wavelength range below 400 nm, was not measured. The examinations were not conducted at a fixed time of the day; instead, they were performed at different times according to participant availability.

Statistical analyses were performed using IBM SPSS Statistics version 24. Based on the spectral transmittance characteristics of the TWC lens and the optical and psychophysical working hypothesis described above, the L-cone contrast sensitivity score was defined as the primary outcome of this study, whereas the M- and S-cone scores were treated as exploratory secondary outcomes. Comparisons between the conditions with and without the TWC lens were conducted using paired *t*-tests, with the significance level set at *p* < 0.05. To provide an omnibus assessment across cone axes, the paired *t*-tests were supplemented with a two-way repeated-measures analysis of variance (ANOVA), with lens condition (without TWC lens and with TWC lens) and cone axis (L, M, and S) as within-subject factors. The main effects of lens condition and cone axis and the lens condition × cone axis interaction were evaluated. Sphericity was assessed using Mauchly’s test, and Greenhouse–Geisser correction was planned if the assumption of sphericity was violated. Because the L-cone-axis score represented the primary hypothesis and the M- and S-cone-axis paired comparisons were exploratory, no multiplicity adjustment was applied to the individual paired comparisons. The repeated-measures ANOVA was used to address the broader omnibus question of whether TWC lens wear affected the cone-axis-specific scores overall and whether the magnitude of this effect differed among cone axes. Given the exploratory pilot design and limited sample size, all findings were interpreted as preliminary. Normality was assessed using the Shapiro–Wilk test of the between-condition differences in cone contrast sensitivity scores for each cone type (*n* = 15; significance level, 0.05). The Shapiro–Wilk test showed no significant deviation from normality for any cone contrast sensitivity score difference (L:W = 0.962, *p* = 0.735; M:W = 0.904, *p* = 0.109; S:W = 0.975, *p* = 0.924).

## 3. Results

**Figure 4:**
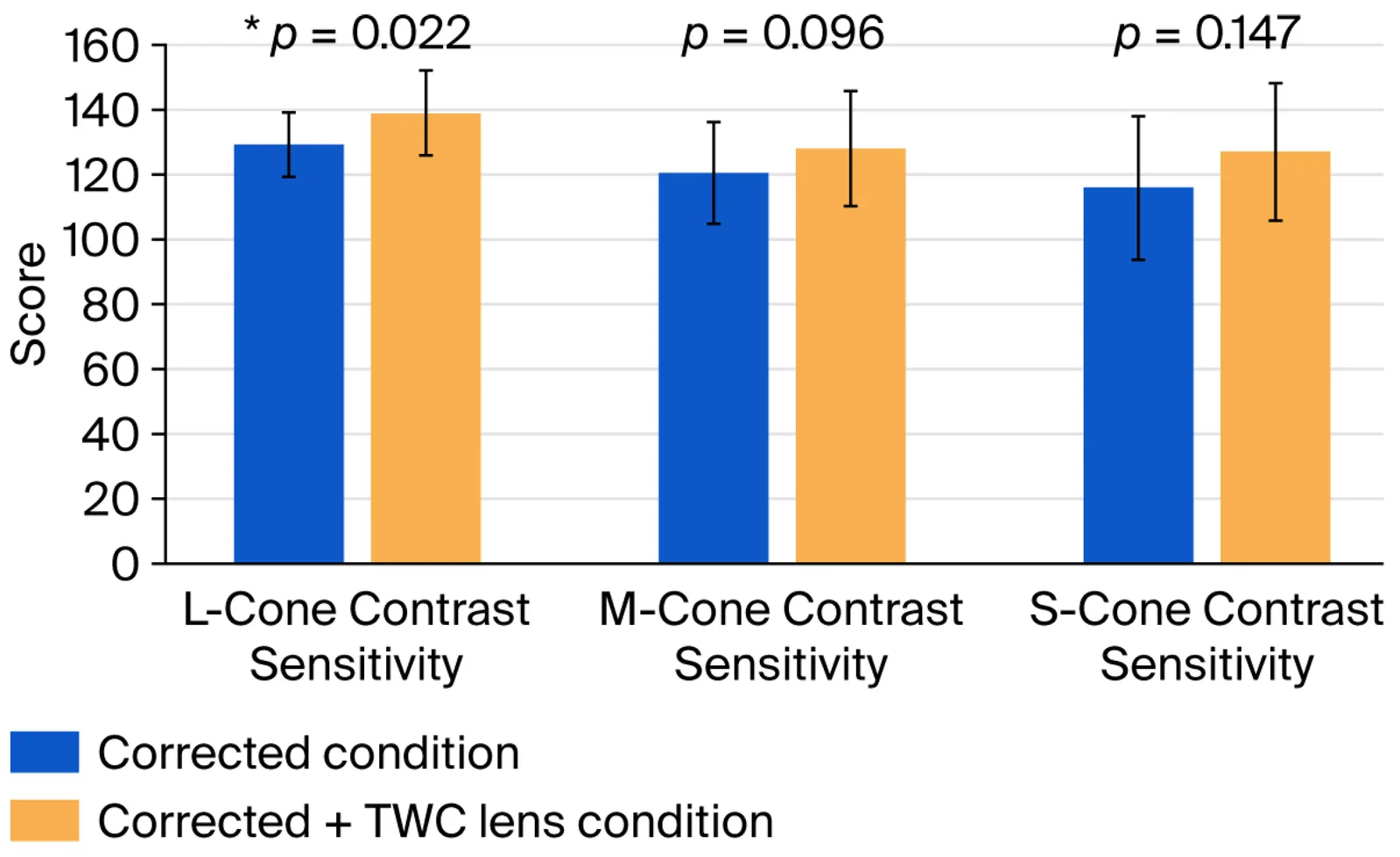
Mean scores obtained with the ColorDx CCT-HD. Blue bars indicate cone contrast sensitivity under the full refractive correction condition alone. Orange bars indicate cone contrast sensitivity under the full refractive correction with the TWC lens condition. * *p* < 0.05.

Figure 4 shows the mean cone contrast sensitivity scores measured under the two conditions: without TWC lens and with TWC lens. For the primary outcome, the L-cone score was 129.4 ± 9.9 in the without TWC lens condition and 139.1 ± 13.1 in the with TWC lens condition, showing a significantly higher value in the with TWC lens condition (paired *t*-test, *p* = 0.022). The mean difference between the two conditions (with minus without) was 9.7, with a 95% confidence interval (95% CI) of 1.7 to 17.8. For the exploratory secondary analysis, the M-cone score was 120.7 ± 15.7 in the without TWC lens condition and 128.2 ± 17.9 in the with TWC lens condition, with no significant difference between the two conditions (paired *t*-test, *p* = 0.096). The mean difference (with minus without) was 7.5, and the 95% CI ranged from −1.5 to 16.6. Similarly, the S-cone score was 116.0 ± 22.2 in the without TWC lens condition and 127.1 ± 21.1 in the with TWC lens condition, with no significant difference between the two conditions (paired *t*-test, *p* = 0.147). The mean difference (with minus without) was 11.1, and the 95% CI ranged from −4.4 to 26.7 (Table 1).

The supplementary two-way repeated-measures ANOVA showed a significant main effect of lens condition (F(1,14) = 6.426, *p* = 0.024, partial η^2^ = 0.315) and a significant main effect of cone axis (F(2,28) = 9.497, *p* < 0.001, partial η^2^ = 0.404). However, the lens condition × cone axis interaction was not significant (F(2,28) = 0.154, *p* = 0.858, partial η^2^ = 0.011), indicating no statistical evidence that the magnitude of the effect of TWC lens wear differed among the L-, M-, and S-cone axes. Mauchly’s test indicated that the assumption of sphericity was not violated for either the cone-axis effect (W = 0.840, *p* = 0.322) or the lens condition × cone axis interaction (W = 0.683, *p* = 0.084).

## 4. Discussion

We investigated the effect of wearing TWC lenses on cone contrast sensitivity in healthy adults. In the primary analysis, the L-cone-axis contrast sensitivity score was significantly higher with the TWC lens than without the TWC lens, whereas the exploratory paired comparisons for the M- and S-cone axes did not reach statistical significance. The present study should be interpreted as an exploratory, cone-axis-focused extension of previous work on wavelength-control lenses. Importantly, the supplementary repeated-measures ANOVA showed a significant main effect of lens condition but no significant lens condition × cone axis interaction. Thus, although the primary L-cone analysis reached statistical significance, the present data provide no statistical evidence that the magnitude of the TWC lens effect differed among the L-, M-, and S-cone axes. The fact that the L-cone paired comparison was statistically significant whereas the M- and S-cone paired comparisons were not should therefore not be interpreted as evidence of an L-cone-specific effect. Rather, the ANOVA results are consistent with an overall lens-associated change in cone-axis-specific contrast sensitivity without demonstrated cone-axis selectivity. Given the small sample size, however, the absence of a significant interaction should not be interpreted as evidence that the effects are necessarily identical across the three cone axes.

The present result can be understood most simply as a filtered-stimulus effect. The ColorDx CCT-HD generates cone-axis-specific chromatic stimuli on the display; however, when the TWC lens is placed in front of the eye, the spectrum reaching the retina is not identical to the nominal display spectrum. Because the TWC lens attenuates light below approximately 420 nm, around 460 nm, and around 585 nm, it may reduce short-wavelength scatter or glare and may also change cone excitation in the wavelength range where L- and M-cone sensitivities substantially overlap. Within established cone-opponent models, L-, M-, and S-cone signals are compared in L+M, L−M, and S−(L+M) channels [2,11,12,13,14,15]. Attenuation around 585 nm may alter the relative spectral excitations of the L- and M-cones and thereby influence the measured cone-axis-specific thresholds. However, the ColorDx CCT-HD measurements along individual L-, M-, and S-cone-isolating axes do not directly assess the postreceptoral L−M opponent mechanism. A more direct assessment of L−M opponency would require chromatic modulation along an L−M opponent direction in color space [13,14]. Accordingly, this interpretation should not be regarded as a validated mechanistic model. The present study provides indirect psychophysical evidence only and did not include spectroradiometric cone-catch calculations, neurophysiological measurements, or independent validation using more precise quantitative color vision testing. Therefore, the higher L-cone-axis score should be interpreted as a lens-dependent change in the measured psychophysical threshold under filtered-stimulus conditions, rather than as evidence of intrinsic enhancement of L-cone function or direct modification of the L−M opponent pathway. This cautious interpretation is consistent with studies using multinotch filters, which have shown that changes in threshold-based indices do not necessarily correspond to subjective improvements in color perception [24,25].

Iizuka and Handa [26] reported that the TWC lens yielded higher contrast sensitivity than neutral-density filters in both young and older adult groups. Although their outcome measures differed from those used in the present study, their findings are consistent with the present results, supporting the possibility that wavelength-selective control exerts effects that cannot be explained solely by reduced light intensity. As the present study targeted middle-aged adults, further investigation is required to clarify the effects of age. The clinical value of this study lies in its potential to provide a cone-axis-resolved framework for evaluating wavelength-control lenses. Although this study was conducted in healthy adults and does not directly demonstrate clinical efficacy in patients, the findings suggest that TWC lenses may influence cone-mediated visual processing. This may be useful for future studies investigating optical interventions for individuals with reduced contrast sensitivity, glare-related symptoms, or color-related visual complaints.

Some limitations should be noted. The risk of bias in this study may have been greatest during the measurement stage, as cone contrast sensitivity was assessed using a psychophysical test based on participants’ responses. Therefore, expectation, response strategy, fatigue, learning effects, and examiner-related bias cannot be completely excluded. The Ishihara pseudoisochromatic plates were used to screen participants for apparent red–green color vision deficiency but do not provide a precise quantitative or diagnostic characterization of subtle color vision phenotypes. More precise color vision tests, such as anomaloscopy, quantitative threshold-based color vision tests, or arrangement tests, were not performed in the present exploratory study; such tests could have provided a more detailed and quantitative characterization of color vision status than Ishihara screening alone. In addition, although the testing order was alternated between participants, potential order and learning or adaptation effects cannot be completely excluded. Furthermore, the small sample size may have introduced selection bias and limited the generalizability of the findings. The L-cone contrast sensitivity score was treated as the primary outcome, whereas the M- and S-cone paired comparisons were exploratory secondary analyses; therefore, no multiplicity adjustment was applied to the individual paired comparisons. To address the broader omnibus question across the three cone axes, these analyses were supplemented with a two-way repeated-measures ANOVA. Although the ANOVA demonstrated a significant main effect of lens condition, the lens condition × cone axis interaction was not significant. Consequently, the present findings do not establish that the effect of TWC lens wear is selective for the L-cone axis. Because this exploratory pilot study included only 15 participants, statistical power to detect an interaction or smaller cone-axis-specific effects was limited. Accordingly, the nonsignificant interaction should be interpreted as an absence of statistical evidence for differential effects across cone axes rather than as evidence that the effects are equivalent. Reproducibility should be examined in future confirmatory studies with larger sample sizes, masking where feasible, more precise characterization of color vision status, and spectroradiometric cone-catch modeling incorporating the display spectrum and lens transmittance. Only the immediate effects of lens use were evaluated, and the relationship between subjective assessments and luminance contrast sensitivity was not examined. In addition, the examinations were not performed at a standardized time of day, which may have introduced variability related to fatigue or diurnal factors. The spectral power distribution of the test environment was not measured, particularly at wavelengths below 400 nm. Because the present measurements were performed using a display under indoor LED illumination, the contribution of light in this short-wavelength UVA region was likely limited. Stockman and Rider have noted that color perception in this spectral region is complicated by ocular fluorescence, particularly lenticular fluorescence, which can be excited by UVA light and emit at visible wavelengths [27]. Therefore, the effects observed under the present indoor testing conditions may not fully represent those under real-world illumination, such as daylight, in which the spectral content below 400 nm may differ. Furthermore, the present study did not include spectroradiometric cone-catch calculations incorporating the display spectrum, lens transmittance, and CIE/Stockman–Sharpe cone fundamentals, nor did it include direct neurophysiological measurements. Therefore, the proposed involvement of L−M opponent mechanisms should be regarded as a theory-based interpretation rather than as a directly demonstrated neural mechanism.

Because this exploratory pilot study was not designed as a confirmatory trial with a formal a priori sample size calculation, the statistical power may have been limited. The observed effect size and confidence intervals obtained in this study may be useful for informing sample size calculations in future confirmatory studies. Future studies should include larger sample sizes, investigate differences across age groups and color vision characteristics, and incorporate subjective assessments and neurophysiological indices to clarify the mechanisms of action of wavelength-control lenses. Overall, the present findings suggest that TWC lens wear may be associated with a change in measured cone-axis-specific psychophysical contrast sensitivity under the present filtered-stimulus conditions. However, this exploratory study does not establish intrinsic enhancement of L-cone function, direct neural modification of cone-opponent processing, or a validated mechanistic model. These results should therefore be regarded as preliminary and require confirmation in future studies incorporating larger sample sizes, masking where feasible, precise color vision characterization, and spectroradiometric cone-catch modeling.

## 5. Conclusions

In this exploratory study, we investigated the effects of TWC lens wear on cone contrast sensitivity in 15 healthy adults. We provide a cone-axis-resolved perspective on the visual effects of TWC lenses, thereby extending the findings of previous research that primarily reported broader measures of visual function. The primary analysis showed a significantly higher L-cone-axis contrast sensitivity score in the TWC lens condition than in the condition without the TWC lens. In the supplementary repeated-measures ANOVA, there was also a significant main effect of lens condition; however, the lens condition × cone axis interaction was not significant. Thus, although the primary L-cone finding was statistically significant, the present data do not provide evidence that the magnitude of the TWC lens effect differs among the L-, M-, and S-cone axes. These findings therefore suggest that TWC lens wear may be associated with a change in measured cone-axis-specific psychophysical contrast sensitivity, but they do not establish a selective enhancement of L-cone function, direct modification of cone-opponent processing, or a validated mechanistic model. Future confirmatory studies incorporating larger sample sizes, masking where feasible, precise color vision characterization, and spectroradiometric cone-catch calculations are required to determine whether this effect is reproducible and to clarify its optical and psychophysical basis.

## Figures and Tables

**Table 1:** Estimated 95% confidence intervals for L-cone, M-cone, and S-cone contrast sensitivity.

	Without TWC Lens Mean ± SD	With TWC Lens Mean ± SD	Mean Difference (With-Without)	Difference 95% CI
L-cone	129.4 ± 9.9	139.1 ± 13.1	9.7	1.7 to 17.8
M-cone	120.7 ± 15.7	128.2 ± 17.9	7.5	−1.5 to 16.6
S-cone	116.0 ± 22.2	127.1 ± 21.1	11.1	−4.4 to 26.7

## Data Availability

The data presented in this study are available upon reasonable request from the corresponding author. The data are not publicly available due to privacy and ethical restrictions.

## Abbreviations

The following abbreviations are used in this manuscript:

L-cones	Long-wavelength-sensitive cones
M-cones	Middle-wavelength-sensitive cones
S-cones	Short-wavelength-sensitive cones
TWC	Three-wavelength-cut
